# P-1028. A Shifting Baseline: Epidemiology and Mortality of Invasive Candidiasis caused by *Candida* Species Recovered at a Single Tertiary Care Center

**DOI:** 10.1093/ofid/ofae631.1218

**Published:** 2025-01-29

**Authors:** James Dickey, Michael Fowler, Eszter Toth, Jesse Raposa, Jose A Vazquez

**Affiliations:** Medical College of Georgia, North Augusta, South Carolina; Medical College of Georgia, North Augusta, South Carolina; Medical College of Georgia, North Augusta, South Carolina; Medical College of Georgia, North Augusta, South Carolina; Medical College of Georgia at Augusta University, Augusta, GA

## Abstract

**Background:**

Invasive candidiasis (IC) refers to a range of infections caused by the fungus *Candida*. Mortality rates from IC are as high as 40% despite antifungal therapy. Candidemia is the most common manifestation and ranks within the top 4 hospital-associated bloodstream infections in the US. In the past, most cases of IC were historically caused by *Candida albicans*. Recently there have been corresponding increases in non-*albicans Candida* species (NAC). This trend is concerning given the observed increases in antifungal resistance among the NAC, such as *C. glabrata*, *C. parapsilosis*, and *C. auris*. Given the high rate of mortality due to IC and increasing rates of antifungal-resistance among the NAC spp., it is critical to provide up-to-date information describing the shift in *Candida* spp.

**Figure:**
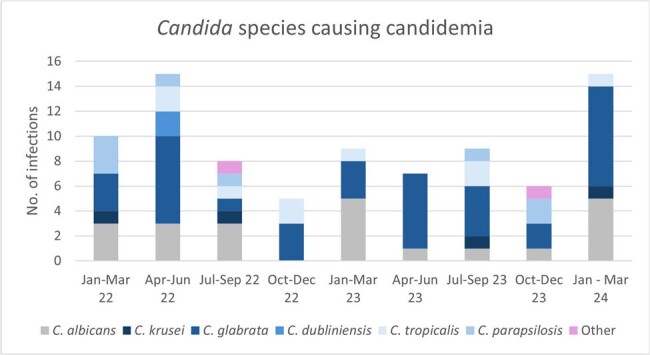

Non-albicans Candida comprised the majority of cases of candidemia, with C. glabrata accounting for the majority of these infections. There was no discernable shift in species distribution across the study period.

**Methods:**

A registry of all *Candida* clinical specimens recovered from anatomically sterile sites at our tertiary care center in Augusta, GA, was developed. Data over a 27-month period was evaluated through a retrospective chart review of all patients with IC. In addition to patient demographics, risk factors, and treatment courses, we analyzed the incidence and the 30 day all-cause mortality of subjects with either *C. albicans* or NAC causing IC.

**Figure:**
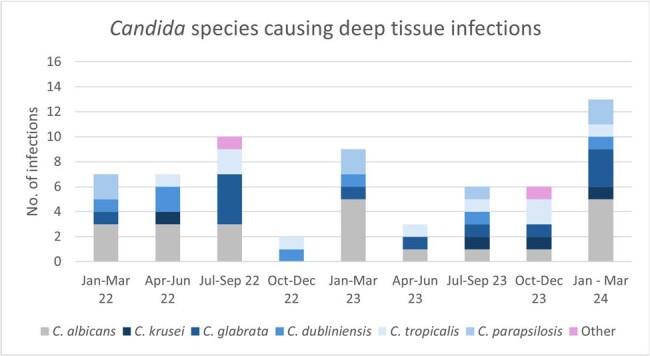

Candida albicans comprised the majority of deep-tissue infections with no discernable shift in species distribution across the study period.

**Results:**

184 cases of IC were identified during this period. NAC spp. comprised the majority of cases of candidemia (64.8%, n=84) while the majority of the deep-tissue infections were caused by *C. albicans* (60%, n=110; Chi-squared, p < 0.001). We identified a notable, non-statistically significant difference in 30-day all-cause mortality between *C. albicans* and all NAC infections. When compared directly, we found a higher 30-day all-cause mortality in infections caused by *C. glabrata* (30.6%, n=49) than *C. albicans* (14.6%, n=89; Chi-squared, p=0.026).

**Conclusion:**

We describe a significant increase in candidemia due to NAC (65%), especially due to C. glabrata. Moreover, there was a slightly higher 30-day mortality due to C. glabrata when compared to C. albicans across all cases of IC. This study establishes a framework to maintain detailed and current, longitudinal evaluations of the epidemiological trends of IC. In addition, knowledge of the shifting epidemiology of IC would also help with the appropriate selection of empiric antifungals and adequate antifungal stewardship.

**Disclosures:**

**All Authors**: No reported disclosures

